# Predicting Outcome in Internet-Based Cognitive Behaviour Therapy for Major Depression: A Large Cohort Study of Adult Patients in Routine Psychiatric Care

**DOI:** 10.1371/journal.pone.0161191

**Published:** 2016-09-12

**Authors:** Samir El Alaoui, Brjánn Ljótsson, Erik Hedman, Cecilia Svanborg, Viktor Kaldo, Nils Lindefors

**Affiliations:** 1 Department of Clinical Neuroscience, Centre for Psychiatric Research, Karolinska Institutet, Stockholm, Sweden; 2 Department of Clinical Neuroscience, Osher Center for Integrative Medicine, Karolinska Institutet, Stockholm, Sweden; 3 Department of Clinical Neuroscience, Division of Psychology, Karolinska Institutet, Stockholm, Sweden; University of Catanzaro, ITALY

## Abstract

**Background:**

Although the effectiveness of therapist-guided internet-based cognitive behaviour therapy (ICBT) for treating depression has been well documented, knowledge of outcome predictors and risk factors associated with lower treatment response is limited, especially when the treatment has been conducted within a naturalistic clinical setting. Identification of such factors is important for clinicians when making treatment recommendations.

**Methods:**

Data from a large cohort (N = 1738) of adult outpatients having been treated with ICBT for depression at an outpatient psychiatric clinic were analysed. A multilevel modelling approach was used to identify patient and treatment variables associated with the speed of recovery during treatment using weekly measurements of the Montgomery Åsberg Depression Rating Scale Self-Rated (MADRS-S).

**Outcomes:**

Adhering to the treatment, perceiving it as credible and working full-time emerged as predictors of a faster pace of recovery and were also associated with a lower level of depression at the end of treatment. Higher pre-treatment depression and sleep problems were associated with a greater improvement rate, but predicted higher depression after treatment. Having a history of psychotropic medication was associated with both slower improvement and higher post-treatment depression.

**Conclusion:**

Perceived credibility of ICBT is a strong predictor of treatment response. Assessing patient beliefs and expectations may be a useful aid for clinicians when identifying those who are more or less likely to benefit from ICBT. Helping patients improve expectations prior to treatment may be an important goal for clinicians during the initial assessment phase.

## Introduction

Major depression is one of the most common mental disorders and is related to lower quality of life and higher risk of suicidal behaviours [1–3]. Cognitive behavioural therapy (CBT) is an effective treatment for depression [4]. To increase accessibility of CBT, online methods of treatment delivery such as therapist-guided internet-based CBT (ICBT) has been recognised as a promising administration format yielding effect sizes at par with conventional face-to-face CBT [5, 6]. However, more knowledge on outcome predictors of ICBT is needed and identifying such determinants can be of high value to clinicians when making treatment recommendations or deciding how to proceed with an ongoing treatment. Given that ICBT is a relatively new treatment format, previous prediction research and risk factors for low response mainly concerns conventional face-to-face CBT. Within clinical and sociodemographic domains, some factors appear to be relatively stable predictors across studies. For example, social support seems to be a major protective factor (e.g. being in a relationship has been associated with a better response [7, 8]) while higher illness severity has been found to predict a worse outcome in some studies [7, 9–11] but not in others [12, 13]. There are fewer predictor studies on ICBT for depression. In regard to the prognostic value of pre-treatment illness severity, findings are inconsistent. In a study from 2004 on outcome predictors of ICBT for depression with 71 participants [14], a weak negative relationship between outcome and those who have had repeated episodes of depression was found, suggesting in line with previous depression research [15] that the level of illness severity is related to treatment outcome. On the other hand, one study found no relation between initial level of depression and outcome [16] and in other studies it has been linked to better response [17, 18]. With regard to substance use, a study from 2014 that included 770 of patients (also included in the present study) treated for depression with ICBT did not detect any effect of problematic substance use on depression outcomes after having excluded patients with substance use disorder [19]. Some pharmacotherapy trials in the treatment of depression have found that substance use disorder was associated with worse outcomes [20, 21] whereas other studies have not identified this relationship [22]. Several studies on ICBT have identified both the level of adherence and treatment credibility as important process measures related to treatment success in self-help interventions such as ICBT [23, 24] where perceived credibility of ICBT [25, 26] and adherence to either face-to-face CBT or ICBT [25–29] has been associated with treatment outcome. A recent study of outcome predictors of ICBT for social anxiety disorder found that patients’ expectations of treatment success and the perceived credibility of ICBT is strongly associated with the rate of improvement, where higher levels of perceived credibility predicts a steeper rate of improvement [30].

This study investigated a large cohort of patients having been treated at a clinical unit for internet treatment, which is part of the Psychiatric Clinic Southwest at Karolinska University Hospital Huddinge, Sweden. The clinical effectiveness of ICBT has been evaluated, not only for depression but also for panic disorder and social anxiety disorder [31–33]. No large-scale study of this size (N = 1738) has previously assessed determinants of symptomatic improvement of ICBT for depression within a clinical psychiatric setting.

## Methods

### Design of the Study

This was a longitudinal study investigating adult patients (N = 1738) who had been treated for depression as part of routine care at the internet psychiatry clinic between 2010 and 2014 were included in the study. Patients could either be referred to the clinic by their general practitioner or register via a self-referral system online. After an online screening procedure, patients were invited to the clinic for a structured diagnostic interview, including the Mini International Diagnostic Interview (MINI) [34], which was performed by a psychiatrist or supervised resident physician. The general guidelines for assessing suitability of ICBT were that patients had to fulfil criteria for major depression according to DSM-IV, be able to write and read in Swedish, not undergo concurrent psychotherapy during the ICBT period and be ≥18 years old and residing in the city county. The following guidelines were followed as exclusion criteria: having severe depression (clinician rated MADRS ≥ 35) and/or moderate to high risk of suicide where monitoring is required; newly (i.e. during the last month) initiated antidepressant medication (ADM); suspected bipolar depression; combinations of comorbidity with high impacts on the complexity of clinical management; low motivation for the internet-format, apathy severe enough to manage self-help, or unable to concentrate on the treatment material; patients with psychosis; in need of drug or alcohol addiction treatment; reading and/or writing difficulties, including language difficulties. Approximately 50% of those who sought treatment were included, and these would typically initiate treatment within one or two days after the psychiatric assessment. The intervention lasted for three months, during which patients were granted access to web-based treatment modules in a step-wise manner and were guided by a licensed psychologist who would provide online support and regular feedback on homework assignments and general progress. Data was collected both during a structured diagnostic interview with a psychiatrist prior to treatment, and from weekly online measurements before, after and during treatment, which have previously been documented as a valid administration format [35]. This research has been approved by the Regional Ethical Review Board in Stockholm, Sweden (no 2011/2091-31/3).

### Primary Outcome Measure

The primary outcome measure was the Montgomery Åsberg Depression Rating Scale Self-Rated (MADRS-S) [36]. MADRS-S measures nine clinical characteristics of depression and the total score scale range is 0 to 54. The test-retest reliability of MADRS-S has been shown to be high (r = .80–.94) [36] and Cronbach’s alpha for the study sample at baseline was .80.

### Candidate predictor variables

A total of 38 candidate predictors were analysed in order to identify those having a significant effect on the rate of improvement over the course of treatment. Due to the large number of candidate predictors, these were grouped and screened in separate domains prior to inclusion in a combined regression model: socio-demographic variables (6 variables), clinical characteristics (11 variables), comorbidity (9 variables), family history of mental illness (10 variables) and treatment related factors (2 variables).

Within the social-demographic domain, the following variables were analysed: age; gender; relationship status (dichotomized as being single or not); level of education rated on a 7-point scale (1 = less than 7–9 years in school; 2 = 7–9 years in school; 3 = incomplete vocational or secondary school; 4 = vocational school; 5 = secondary school; 6 = university, started but not completed studies; 7 = completed university studies); employment status dichotomized as working full-time or not; and having children.

The clinical characteristics domain included the Clinical Global Impression–Severity Scale [37] (CGI-S) which was rated on a 7-point scale; the AUDIT [38] and the DUDIT [39] which were used to screen for alcohol problems or drug misuse; sleep problems measured with the insomnia severity index [40, 41], duration of illness (years since onset), history of psychotropic medication, history of inpatient psychiatric care, history of depression, attempted suicide, currently on medication, and currently on psychotropic medication.

Presence (yes/no) of any comorbid illness was assessed during the diagnostic interview prior to treatment using the MINI. These included panic disorder, agoraphobia, social anxiety disorder, mild depressive episode, moderate depressive episode, severe depressive episode, recurrent mild depressive episode, recurrent moderate depressive episode, recurrent severe depressive episode, recurrent depression without current symptoms and dysthymia.

Having a family history of mental illness was also assessed during the diagnostic interview prior to treatment. The following set of variables were assessed: having a family history of dependence / substance abuse, bipolar disorder, depression, minor depression, neuropsychiatric condition, anxiety, panic disorder, psychosis, social anxiety disorder, social anxiety disorder-like symptoms, suicide attempts or suicide completed.

Finally, treatment process factors included two variables. First, a measure of perceived treatment credibility measured during the second week in treatment, measured with the credibility/expectancy scale [42] where patients’ attitudes to the credibility of the treatment and expectancy regarding treatment effectiveness were rated on a 10-point scale (0 = not at all to 10 = very much). Second, treatment adherence was measured at post-treatment, reflecting the degree of use of the ICBT program operationalized as the total number of activated treatment modules.

### Statistical Analyses

Longitudinal multilevel modelling of repeated measurements was used to identify the variables that had a significant influence on the rate of improvement between pre- and post-treatment. These models were built using the linear mixed-effects models procedure in SPSS version 22. Multilevel modelling was considered to be a suitable analytical approach for several reasons. For example, a multilevel model is able to make use of all available data, rendering it a full intent to treat analysis, thus not omitting cases with incomplete observations from the analysis (where occurrence of missing values lead to more missing values, e.g. through list-wise deletion). Second, it takes into account the hierarchical nature and dependency of the data (i.e., repeated measurements are nested within patients). Of primary interest was the effect of predictor variables on the rate of change (i.e. the slope of the regression line).

Due to the relatively large amount of candidate predictors, all variables were first grouped into separate domains as described above. Within each domain, a separate multilevel model was developed in a stepwise manner where variables were successively evaluated as to whether they significantly had an effect on the rate of symptomatic improvement, a procedure previously adopted by Fournier et al. [43] and Amir et al. [44]. In the first step (Step 1), a model which included all candidate predictors within a specific domain, and their interaction with time (predictor × time), was tested. Only those variables that had an interaction with time at the *p* < .20 level were included in a new model (Step 2). In Step 3, those variables that met the *p* < .10 level were kept and finally assessed in Step 4, at the *p* < .05 level. This procedure was repeated for all predictor domains. Finally, all predictors that were significant at the *p* < .05 level were combined in a final model which consequently tested the predictive value of each predictor from all domains while controlling for the effects of the other variables.

All models contained two levels, where the first level was the repeated MADRS-S measurements from each patient, and the second level consisted of the patients with their individual characteristics. Since the primary interest in this study was the total change over the course of treatment (i.e. improvement between pre- and post-treatment assessment, as opposed to weekly change) and status at post-treatment, the time variable was re-centred to post-treatment (i.e. the value at the intercept reflects level of depression at post-treatment) and re-coded the values to range between -1 and 0. This approach has previously been adopted by Smits and colleagues [45]. Also, all predictor variables were standardized prior to analysis in order to facilitate comparison of slope coefficients for the different predictors [45].

### Ethics Statement

The study, including its consent procedure, was approved by the Regional Ethical Review Board in Stockholm, Sweden (no 2011/2091-31/3). This research was conducted as a retrospective cohort study; therefore, active informed consent was not required by the ethics committee. However, all participants were provided with a choice of participation through an opt-out methodology. Since passive consent does not violate the option of providing choice and increases the likelihood of having a representative sample, this approach is considered to be an efficient procedure for registry data [46, 47].

## Results

### Sample Description

Patient characteristics are presented in Table 1.

**Table 1:** Descriptive statistics of patients treated for depression.

	Statistic
**Socio-demographic variables**	
Age, mean (s.d.)	37.73 (12.08)
Male gender, %	33%
Single, %	41%
Have children, %	47%
Working full-time, %	52%
Working part-time, %	12%
Level of education, mean (s.d.)^a^	5.94 (1.27)
**Clinical variables**	
MADRS-S at baseline, mean (s.d.)	22.35 (6.69)
MADRS-S at post-treatment, mean (s.d.)	13.22 (8.37)
AUDIT, mean (s.d.)	5.24 (4.84)
DUDIT, mean (s.d.)	0.35 (1.58)
Years since onset of symptoms, mean (s.d.)	10.30 (10.20)
GAF, mean (s.d.)	61.50 (7.41)
Insomnia Severity Index (s.d.)	12.80 (5.86)
CGI-S Global Severity, mean (s.d.)	3.80 (2.48)
Currently on psychotropic medication, %	72%
History of depression, %	69%
History of inpatient psychiatric care, %	6%
History of psychotropic medication, %	44%
**Comorbid diagnoses**	
Comorbid panic disorder, %	6%
Comorbid agoraphobia, %	5%
Comorbid social anxiety disorder, %	17%
**Family history of mental illness**	
Family history of dependence / substance abuse, %	18%
Family history of bipolar disorder, %	6%
Family history of depression, %	30%
Family history of minor depression, %	19%
Family history of neuropsychiatric condition, %	4%
Family history of anxiety, %	5%
Family history of panic disorder, %	3%
Family history of psychosis, %	4%
Family history of social anxiety disorder, %	1%
Family history of social anxiety disorder-like symptoms, %	2%
Family history of suicide attempts, %	7%
Family history of suicide completed, %	10%
**Treatment-related factors**	
Adherence (no. of activated treatment modules), mean (s.d.)	7.21 (3.09)
Treatment credibility scale (s.d.)	33.03 (8.47)

a. Level of education was rated on a 7-point scale (1 = less than 7–9 years in school; 2 = 7–9 years in school; 3 = incomplete vocational or secondary school; 4 = vocational school; 5 = secondary school; 6 = university, started but not completed studies; 7 = completed university studies).

### Attrition and Adherence

Table 2 presents the number of observations for each measurement occasion of the outcome variable (MADRS-S) used in the analyses.

**Table 2:** Number of observations and means for each measurement occasion.

	N	Mean	SD
MADRS-S pre-treatment	1738	22.35	6.69
MADRS-S week 1	1517	20.16	7.09
MADRS-S week 2	1500	18.95	7.47
MADRS-S week 3	1447	17.98	7.78
MADRS-S week 4	1390	17.33	7.85
MADRS-S week 5	1344	16.56	7.84
MADRS-S week 6	1307	15.98	8.02
MADRS-S week 7	1229	15.17	7.83
MADRS-S week 8	1202	14.49	7.96
MADRS-S week 9	1121	13.57	7.83
MADRS-S week 10	1083	13.11	7.86
MADRS-S post-treatment	1447	13.22	8.37

As shown in Table 2, 1447 patients provided post-treatment scores on the main outcome measure, whereas 291 patients had missing data on this measurement occasion. There was a significant difference in baseline level of depression between patients with no missing data at post-treatment (*M* = 22.14, *SD* = 6.51) and patients with missing post-treatment scores (*M* = 23.42, *SD* = 7.43) conditions; *t*(1736) = -2.99, *p* < .05.

The mean level of adherence was 7.21 (SD = 3.09) activated treatment modules out of 10. The median level was 8 with and the interquartile range (IQR) was 5.

### Predictors of symptomatic change

**Fig 1:**
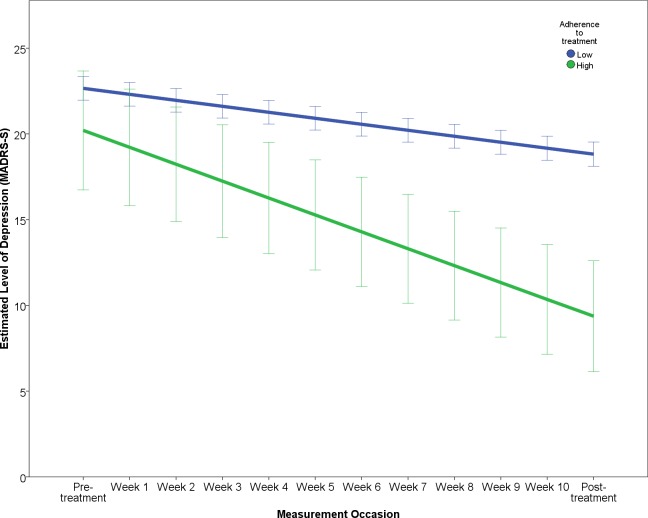
Effect of adherence on change in depression.

**Fig 2:**
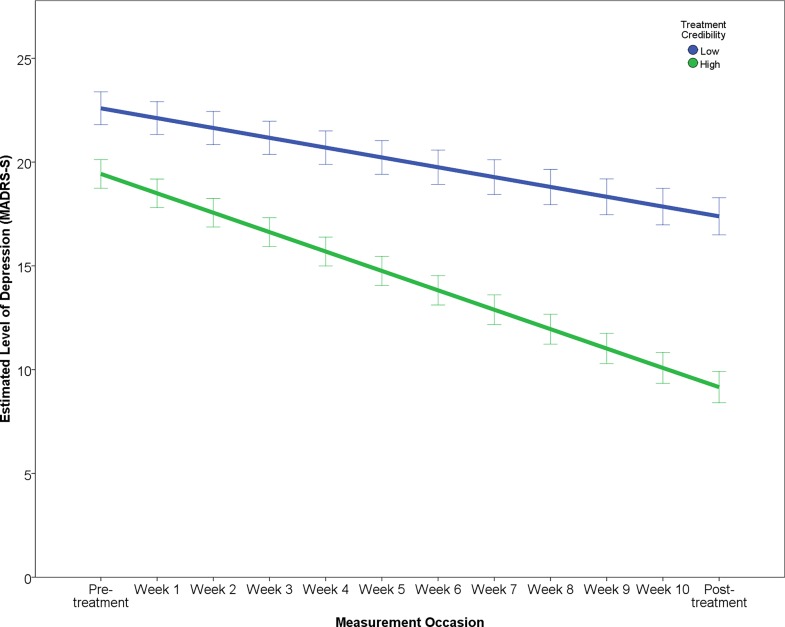
Effect of treatment credibility on change in depression.

**Fig 3:**
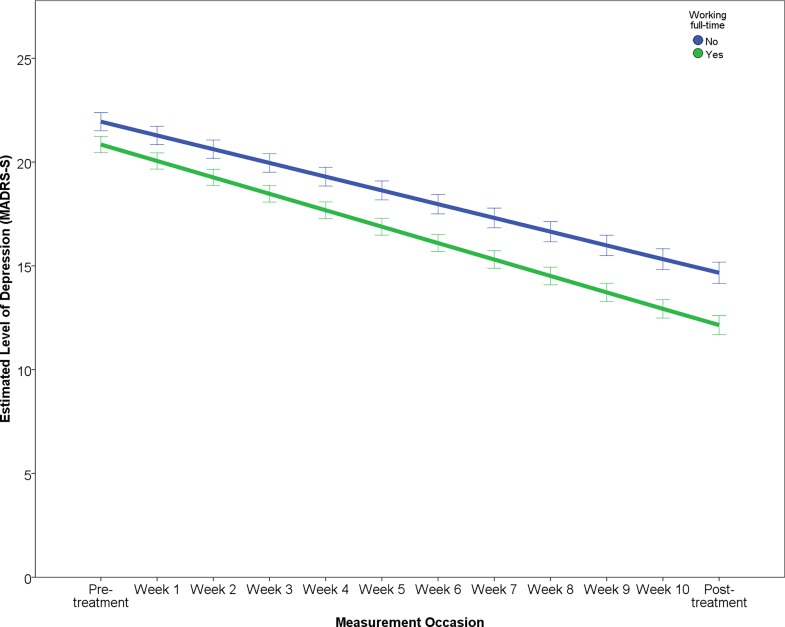
Effect of employment status on change in depression.

**Fig 4:**
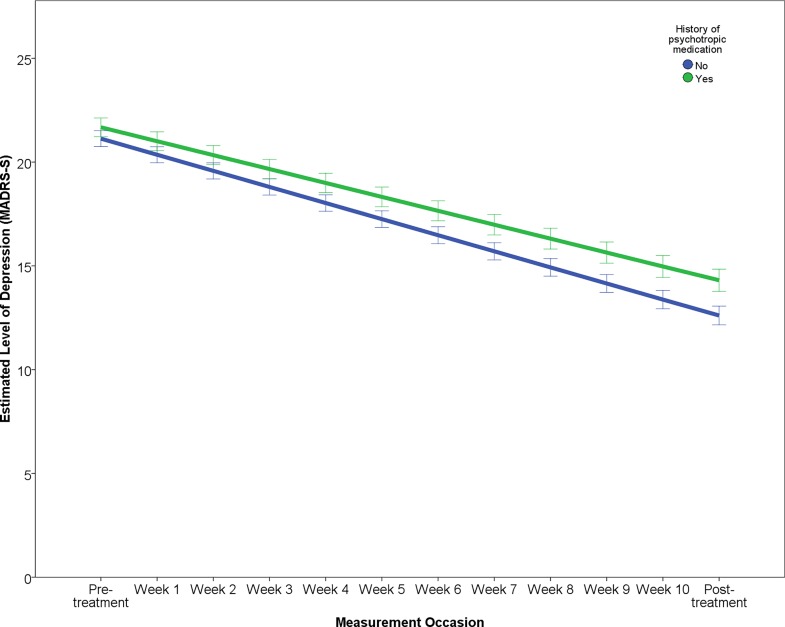
Effect of having a history of psychotropic medication on change in depression.

**Fig 5:**
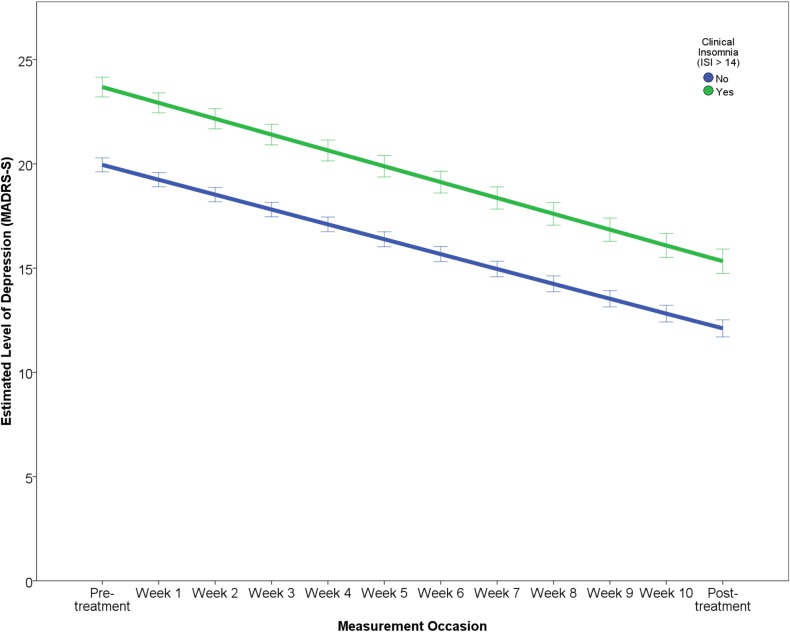
Effect of sleep problems on change in depression.

The results of the final multilevel model predicting symptomatic improvement are presented in Table 3. The outcome variable “Level of depression” reports the estimated change over the study period in terms of MADRS-S scores, predicting that patients will improve by about 7.89 points and that after treatment patients will have an estimated level of depression of about 13.51 points on the MADRS-S. The effect of each predictor on the rate of improvement (i.e. interaction of predictor × time) is illustrated in Figs 1–5. In order to depict the effects of the predictors, continuous predictor variables were categorized into a “high” and “low” group, where a “high” score was operationalized as one standard deviation above the mean and a “low” score as one standard deviation below the mean. The division in groups regarding the levels “high” and “low” of the predictors were used for the graphic illustrations. In the analysis, however, all data from all participants was used.

**Table 3:** Effects of socio-demographic, clinical and treatment-related variables on depression.

	Effect on rate of improvement		Effect on post-treatment score	
	*Coefficient* (S.E.)^a^	*p*	*Coefficient* (S.E.)^b^	*p*
**Outcome**				
Level of depression (MADRS-S)	-7.89 (0.18)	***	13.51 (0.19)	***
**Predictors**				
**Socio-demographic variables**				
Working full-time	-0.45 (0.18)	**	-0.83 (0.19)	***
Female	-0.26 (0.18)		0.17 (0.19)	
Single	0.09 (0.18)		0.61 (0.19)	***
**Clinical variables**				
Insomnia severity (ISI)	-0.62 (0.18)	***	1.54 (0.19)	***
History of psychotropic medication	0.51 (0.18)	***	0.52 (0.19)	**
Drug use (DUDIT)	0.25 (0.18)		0.29 (0.18)	
**Treatment-related variables**				
Adherence	-2.29 (0.21)	***	-2.75 (0.22)	***
Treatment credibility	-1.11 (0.19)	***	-1.94 (0.20)	***

Note. Significant effects on outcome are denoted as

***, *p* < ∙001

**, *p* < ∙01 and

*, *p* < ∙05.

^a^ Values represent standardized beta coefficients predicting the effect on the rate of change (slope) in self-rated symptoms of depression (MADRS-S) over the full course of treatment. Negative values indicate greater change during treatment.

^b^ Values represent standardized coefficients predicting the effect on MADRS-S at post-treatment. Negative values indicate lower estimated post-treatment scores.

According to the final model, working full-time, high treatment adherence and ratings of perceived treatment credibility predicted both greater rate of improvement as well as lower post-treatment level of depression (Figs 1–3).

Initial level of depression predicted a greater rate of improvement but also a higher level of post-treatment scores. Neither gender nor relationship status were related to individual differences in rate of improvement, although being single was associated with a higher post-treatment level of depression.

Within the domain of clinical characteristics, patients with a history of psychotropic medication showed both a slower rate of improvement as well as a higher level of post-treatment depression (Fig 4). Increasing severity of self-reported sleep problems prior to treatment was related to a faster rate of improvement (Fig 5) but also higher post-treatment depression.

Neither the presence of any of the co-morbid diagnoses tested nor having a family history of mental illness was related to the rate of improvement.

## Discussion

### Main Findings

The aim of this study was to identify patient and treatment characteristics associated with symptomatic improvement in a large cohort of depressive patients after ICBT within a naturalistic clinical setting. The degree to which patients adhered to the treatment had the strongest overall relationship with the rate of improvement. The average adherence in the study was about 70%, which is similar to the median adherence of 69% reported in a recently published review of ICBT trials of treating depression [48]. However, since adherence only can be fully measured after the intervention, it might be of more use to clinicians to identify pre-treatment variables–or process variables measured early in treatment–that have prognostic value. Thus, treatment credibility was identified as the strongest *prognostic* variable, an observation in line with previous research having identified a significant association between treatment credibility and the effectiveness of ICBT [25, 26]. The findings of the relationship between perceived credibility of the treatment and patients’ expectancies–including expectations of the effects of treatment–could indicate the involvement of a placebo effect. The question then concerns whether the observed treatment response is caused by the intervention or by patient’s expectations. However, a patient is not a passive receiver of therapy, but rather actively interacting with the treatment content to produce changes in behaviour and cognitions. It is logical then, that expectations may have a moderating effect on the relationship between being exposed to ICBT and responding to treatment; a placebo effect could therefore be regarded as an integral part of every therapeutic intervention, indicating a central role of perception in treating depression. It may be noted that a meta-analysis of the placebo effect in antidepressant trials reported that the placebo effect accounted for as much as 68% of the treatment response [49]. In the case of ICBT, it is reasonable to assume that adherence and credibility are correlated, and that the relationship between credibility and outcome may be mediated by adherence. Further, although having a correlation between the credibility and outcome might not explain an overall placebo effect, it could still be that those who have low credibility make proper improvements.

We found no significant relationship between gender and rate of improvement, which is in line with previous predictor studies on ICBT for depression where no association could be detected between gender and therapeutic outcome [14, 50]. Since this study had a relatively large cohort, it might have increased the possibility of identifying this relationship.

Also in line with research on conventional treatments for depression and the protective effect of social relationships (e.g. being married has been linked to a better outcome [7, 8]), we found that being single was associated with a worse end state. Further, having a history of psychotropic medication predicted both a worse end state and a slower improvement rate, whereas working full-time emerged as a protective factor. Although sleep problems were associated with a slightly steeper improvement rate (perhaps since addressing sleep problems is addressed during treatment in a module specifically devoted to this problem area), we also found that these patients were more depressed after treatment which is in line with previous findings that patients suffering from both conditions more seldom achieve sufficient relief [51]. In line with a previous ICBT study on the predictive role of alcohol use on treatment outcome [19], our multilevel model found no significant relationship between alcohol use and rate of improvement when controlling for other factors within the same model. Difficulties in detecting such a relationship might be partly explained by the fact that patients who have a significantly problematic alcohol use were generally excluded from the treatment and thus from the study sample.

### Study Limitations

The present study was not a randomized controlled trial; therefore, we could not compare the proportion of patients with non-response with a control group of patients receiving no treatment for depression. However, there are a number of studies on the natural course of depression who report that a significant proportion of patients within primary care generally do not recover spontaneously [52, 53]. Also, in a recent study on ICBT for depression, patients receiving treatment as usual (TAU) improved significantly less compared to the ICBT group [54]. Another limitation concerns the external validity, since approximately 50% of patients who sought treatment were excluded from ICBT treatment; the results are therefore based on predictor variables of a biased and non-random sample which may have implications for which populations these results may be generalized to.

Some of the exclusion criteria such as low motivation, severe apathy, and difficulty concentrating are related to basic criteria for diagnosing depressive conditions. It may seem counter-intuitive that some of these factors were used to diagnose patients and to exclude patients at the same time. However, to propose ICBT it is essential with an overall assessment of the ability and motivation to manage the specific treatment format. Hence, once a depressive diagnosis has been established, the clinician must also make an informed overall evaluation regarding the severity of the condition and the ability of the patient to manage the treatment. Mostly, exclusion of depression patients is made on the same basis as for face-to-face CBT, i.e. when non-psychological treatment is more indicated (high severity), or the patient has other problems that need to be investigated more (e.g. suspicious bipolar II disorder) or specifically treated (e.g. PTSD). However, there is a component for the position to ICBT that places additional demands on the patient, namely the ability to concentrate on reading and understanding written text. When this ability is unclear we often have patient samples read the paper module or read the first section of the module to see whether the patient regards the treatment form as suitable.

We also found a significant difference in baseline level of depression between patients who dropped out versus those who provided post-treatment scores on the MADRS-S. However, the mean difference was only -1.3 (95% CI: -2.13 to -0.44) and we believe that the assumption used in the multilevel models (i.e. that the data is missing at random, MAR, not missing completely at random, MCAR) is valid and that this difference is ignorable and have not likely biased the predicted estimates. Further, this analysis indicates MAR on the grounds that we found an observed variable that significantly explains missingness. Had the difference been greater, it had still been in accordance with the MAR assumption as long as the observed variable (pre-value) is included in the analysis model used in the study (which it is in the present study).

### Conclusion

Several factors of importance for ICBT treatment outcome have been identified. Results indicate that assessing patients’ perceived treatment credibility of ICBT may be highly informative with regard to probable treatment response. These findings may provide clinicians with supportive information when considering patients for ICBT.
